# Increased Systemic Antioxidant Power Ameliorates the Aging-Related Reduction in Oocyte Competence in Mice

**DOI:** 10.3390/ijms222313019

**Published:** 2021-12-01

**Authors:** Sydney L. Lane, Jason C. Parks, Jennifer E. Russ, Shaihla A. Khan, William B. Schoolcraft, Ye Yuan, Mandy G. Katz-Jaffe

**Affiliations:** Colorado Center for Reproductive Medicine, Lone Tree, CO 80124, USA; SydneyLane48@gmail.com (S.L.L.); jparks@colocrm.com (J.C.P.); jeruss@umich.edu (J.E.R.); shaihla.khan@googlemail.com (S.A.K.); billsgrants@colocrm.com (W.B.S.); YYuan@FLColo.com (Y.Y.)

**Keywords:** oxidative stress, antioxidants, aging, infertility, oocyte competence

## Abstract

Ovarian aging is associated with elevated oxidative stress and diminished oocyte developmental competence. We aimed to determine the impact of systemic antioxidant treatment in aged mice. Female outbred CF-1 mice were aged for 9 months prior to an 8-week 45 mg *Euterpe oleracea* (açaí) daily supplement. The açaí treatment induced a threefold increase in serum antioxidant power (FRAP) compared to both young and aged mice (*p* < 0.0001). Compared to young mice, aged mice had fewer oocytes and reduced blastocyst development (*p* < 0.0001); açaí did not affect the oocyte numbers, but improved blastocyst formation (*p* < 0.05). Additionally, açaí alleviated the aging-related decrease in implantation potential (*p* < 0.01). The aged mice showed evidence of elevated ovarian ER stress (increased whole-ovary *PDIA4* expression, granulosa cell and oocyte *GRP78* expression, and oocyte PDIA4 protein), reduced oocyte mitochondrial quality (higher PRKN activation and mitochondrial DNA oxidative damage), and dysregulated uterine glandular epithelium. Antioxidant intervention was sufficient to lessen these effects of ovarian aging, likely in part by the upregulation of NRF2. We conclude that açaí treatment is a promising strategy to improve ER and mitochondrial function in the ovaries, thereby ameliorating the decreased oocyte competence that occurs with ovarian aging.

## 1. Introduction

Inferior oocyte developmental competence is associated with advanced maternal age [1,2]. As women increasingly postpone pregnancy into their thirties and forties [3], poor oocyte quality is a primary concern in infertility treatment. Elevated rates of aneuploidy occur in aged oocytes, due largely to meiosis I (MI) errors [4,5], but even in the absence of chromosomal abnormalities, aged oocytes are less likely to undergo fertilization and blastocyst formation [6,7].

Ovarian aging is associated with redox imbalance, specifically a shift toward pro-oxidant status, with both reduced antioxidant capacity and more reactive oxygen species (ROS) generated [8,9,10]. Important processes in the ovary—such as MI progression, follicular atresia, and luteal regression—depend on physiological ROS as signaling molecules [11,12]; however, pathologically high ROS levels are detrimental to normal ovarian function and the ultrastructural characteristics of oocytes [13,14].

The mechanisms of aging-related ROS-induced damage include dysfunctional mitochondria, which reduces ATP production and results in further ROS production [15]. Proteins, lipids, and DNA—both nuclear and mitochondrial—are susceptible to ROS-induced damage, causing deficiencies in many cellular processes, including redox maintenance [16]. Misfolded and oxidatively damaged proteins accumulate, leading to endoplasmic reticulum (ER) and mitochondrial stress, and the activation of the unfolded protein response (UPR).

The increased oxidative stress observed with aging or other pathologies represents a target for therapeutic intervention. The restoration of redox balance may result in improved cellular function and clinical outcomes. Indeed, antioxidant supplements have been utilized clinically for many diseases, including cancer, neurodegeneration, diabetes, and chronic obstructive pulmonary disease [17,18]. Recent studies of antioxidant treatment with melatonin or Coenzyme Q10, which improves mitochondrial function, reported improved follicular fluid oxidative balance and readouts of ovarian function, including the rate of blastocyst development [19,20,21,22].

Here, we utilize the naturally occurring açaí berry, which has a proven high antioxidant capacity. The polyphenolic compounds in açaí—including anthocyanins, phenolic acids, and flavonoids—scavenge ROS directly, such that genetic and metabolic variability in patients are unlikely to influence its effectiveness [23,24]. Açaí intervention has been shown to reduce oxidative stress in animal models of high-fat diet-induced obesity, renovascular hypertension, and acute colitis, among others [25,26,27]. Our preliminary work investigating the açaí berry in mice revealed positive effects on ovarian and oocyte signaling, with upregulated antioxidant signaling and lower markers of ER stress and apoptosis [28]. Clinically, the use of this antioxidant supplement prior to egg retrieval resulted in a high live birth rate following single euploid frozen embryo transfer in patients with prior in vitro fertilization (IVF) failures [28].

In the current study, mice were naturally aged for 9 months prior to 8 weeks of dietary supplementation with açaí berry pulp. We hypothesized that açaí supplementation in aged mice improves the pro- to antioxidant balance, and prevents the aging-associated decline in oocyte quality. We found that antioxidant treatment in aged mice did not impact the oocyte number, but was sufficient to improve markers of oocyte quality, including the rate of blastocyst development and uterine implantation in untreated aged recipient mice. This correlated with reduced markers of ER stress and mitochondrial dysfunction in the ovary and oocyte.

## 2. Results

### 2.1. Reproductive Outcomes

**Figure 1:**
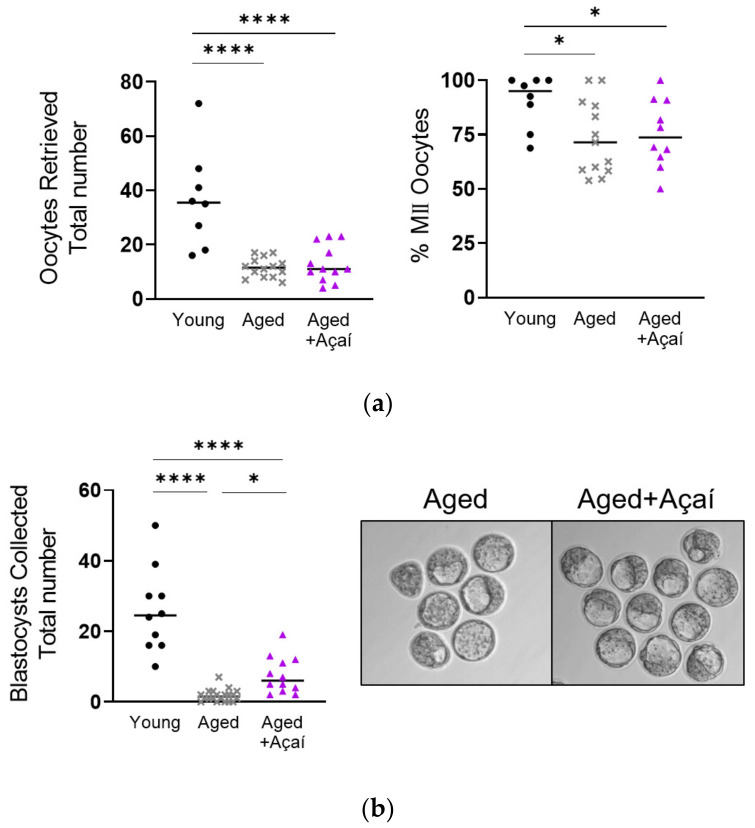
Effects of age and açaí treatment on oocyte and blastocyst development. (**a**) Numbers of total oocytes retrieved from superovulated mice, and the proportion that were MII vs. degenerate (n = 8 young, 14 aged, 12 aged + açaí). (**b**) Numbers and representative images of D4 embryos collected from superovulated, bred mice (n = 10 young, 20 aged, 12 aged + açaí). The data were analyzed by the Kruskal–Wallis test with Dunn’s multiple comparisons when the overall effect *p* < 0.05. * *p* < 0.05, **** *p* < 0.0001.

Açaí supplementation was well tolerated by the aged mice, and did not affect weight gain or fluid intake (*p* = 0.68 and *p* = 0.65, respectively). Superovulated control aged and açaí-treated aged mice produced fewer oocytes than young mice (both *p* < 0.0001); there was no difference between the aged groups (*p* > 0.99) (Figure 1a). The three cohorts produced a similar number of degenerate oocytes (*p* = 0.84), such that the proportion of metaphase II (MII) oocytes was lower in both aged groups compared to the young mice (both *p* < 0.05).

Of the superovulated control aged mice that successfully bred with fertile males, only 50.0% (20/40) had in-vivo-developed D4 embryos, compared to 100% (13/13) of the young mice (*p* < 0.001) and 85.7% (18/21) of the açaí-treated aged mice (*p* < 0.05). In order to eliminate the chance that bred, plugged mice did not successfully breed, the numbers of blastocysts were analyzed only for the mice with detectable embryos of any stage. Of the mice with detectable D4 embryos, the aged mice had significantly fewer blastocysts than the young (1.75 ± 0.38 vs. 25.9 ± 3.8; *p* < 0.0001) or açaí-treated aged mice (7.58 ± 1.51; *p* < 0.05) (Figure 1b). The numbers of morulae and degenerate embryos did not differ between the groups (*p* = 0.11 and *p* = 0.74, respectively).

Because similar numbers of oocytes were retrieved from the control aged and açaí-treated aged mice, but significantly more embryos were collected from açaí-treated mice, the rate of blastocyst formation per oocyte ovulated was higher in the açaí-treated than the control aged mice (89.6 vs. 17.8%, respectively).

**Figure 2:**
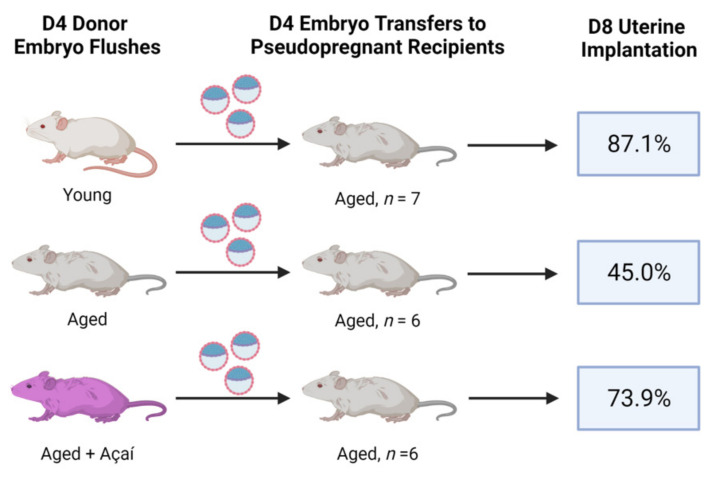
Embryo transfer outcomes in aged recipient mice. Embryos were collected from superovulated, bred mice on gestational day 4 (D4) and transferred into pseudopregnant D4 mice. The implantation rates were determined on D8.

Expanded D4 blastocysts from young, control aged, and açaí-treated aged mice were transferred into pseudopregnant aged mice. The embryos from the control aged donors were less likely to implant in the recipient aged mice compared to embryos from young (relative rate 0.52 [95% CI: 0.29–0.79]; *p* < 0.01) or açaí-treated aged donors (relative rate 0.61 [95% CI: 0.34–1.01]; *p* = 0.068) (Figure 2). The embryos from the young and açaí-treated aged donors were equally likely to implant in the recipient aged mice (relative rate 1.18 [95% CI: 0.90–1.66]; *p* = 0.29).

### 2.2. Serum Changes following Aging and Antioxidant Supplementation

**Figure 3:**
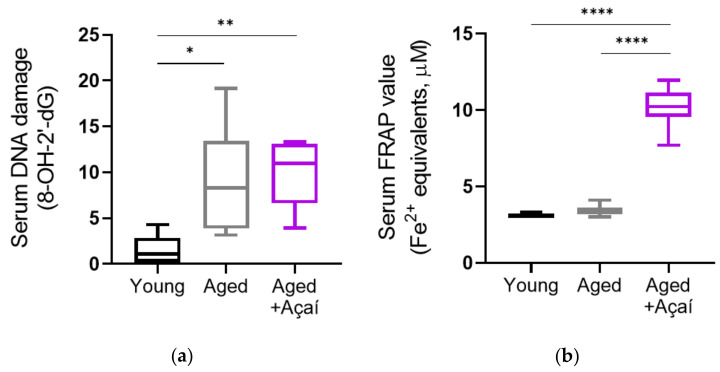
Systemic evidence of an altered redox state. (**a**) Levels of the DNA oxidative damage marker 8-OH-2′-dG in the serum from non-pregnant female mice (n = 6 young, 8 aged, 8 aged+ açaí). (**b**) Ferric reducing antioxidant power (FRAP) values indicative of the total antioxidant power in the serum from non-pregnant female mice (n = 6 young, 9 aged, 9 aged+ açaí). The data were compared using ordinary one-way ANOVA with Tukey’s multiple comparisons when the overall effect *p* < 0.05. * *p* < 0.05, ** *p* < 0.01, **** *p* < 0.0001.

The serum levels of 8-hydroxy-2′-deoxyguanosine (8-OH-2′-dG), an oxidative stress-induced DNA damage marker, were elevated in the control aged mice compared to the young mice (*p* < 0.05) (Figure 3a). Antioxidant treatment did not prevent this effect, as the serum 8-OH-2′-dG levels in the açaí-treated aged mice were higher than those in the young mice (*p* < 0.01), and were not different from the control aged mice (*p* = 0.92).

The serum ferric reducing antioxidant power (FRAP) values, which are indicative of systemic antioxidant power, were similar in young and control aged mice (*p* = 0.74) (Figure 3b). The açaí treatment in aged mice significantly increased the serum FRAP values compared to both the young and control aged mice (both *p* < 0.0001).

### 2.3. Effects of Aging and Açaí on Ovarian ER Stress

**Figure 4:**
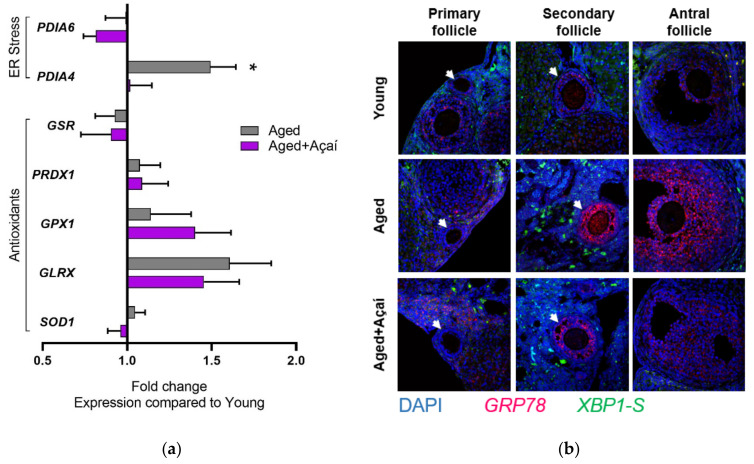

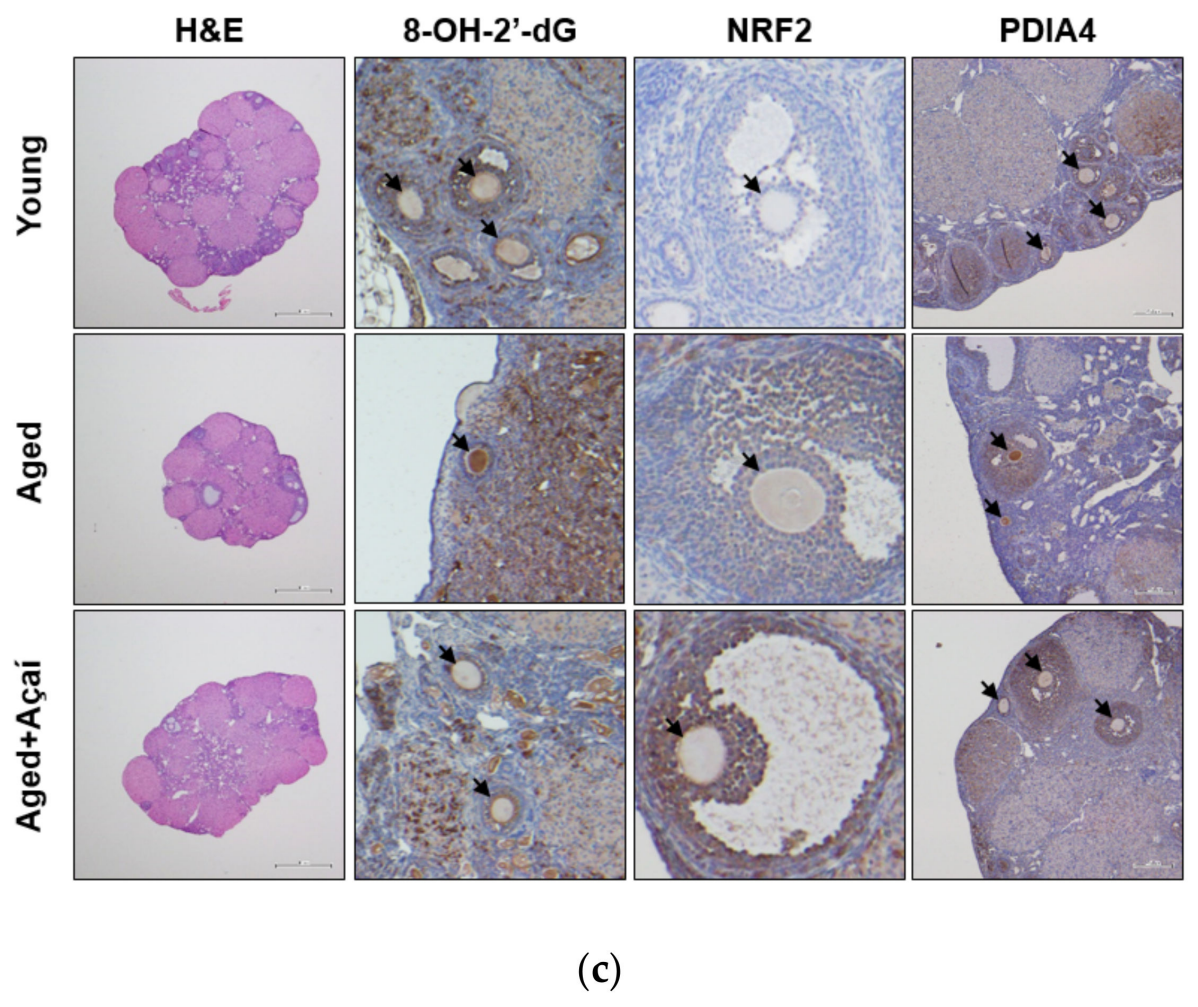
Biochemical changes in the ovary. (**a**) Whole-ovary gene expression of ER stress and antioxidant-related genes (n = 10 mice per group). (**b**) RNA in situ hybridization of ovarian sections (n = 5 mice per group). (**c**) Immunohistochemistry of ovarian sections for the localization of DNA damage marker 8-OH-2′-dG and proteins NRF2 and PDIA4 (n = 5 mice per group). The black arrows indicate oocytes. Scale bars: 1 mm for the HE images and 200 μm for the IHC images; all of the IHC images are presented at the same magnification. The gene expression data were analyzed by ordinary one-way ANOVA with Tukey’s multiple comparisons when the overall effect *p* < 0.05. * *p* < 0.05 compared to young mice.

The expression of the ER stress-related gene *PDIA4* was higher in the whole ovary from the control aged mice compared to the young mice (*p* < 0.05); this effect was prevented by açaí treatment (Figure 4a). The expression of the antioxidant genes *SOD1*, *GLRX*, *GPX1*, *PRDX1*, and *GSR* was not significantly different among the three groups.

Endoplasmic reticulum stress was further evaluated using RNA in situ hybridization of ovarian sections. The expression of the ER stress marker *GRP78* was not different among the three groups in the primary follicles (Figure 4b). In the secondary and antral follicles, *GRP78* expression was elevated in the cumulus and granulosa cells in the aged mice; this was not observed following açaí treatment. The spliced, activated form of the ER stress marker *XBP1* (*XBP1-S*) was expressed in the thecal and cortical stromal cells of the ovary, and was not different between the groups.

Immunohistochemical localization of the oxidative stress-induced DNA damage marker 8-OH-2′-dG revealed high DNA damage in control aged ovaries, with marked expression in oocytes (Figure 4c). In contrast, the expression of the DNA damage marker was lower overall and not detectable in oocytes in ovaries from young and açaí-treated aged mice. The expression of the transcription factor NRF2 was slightly elevated in cumulus, granulosa, and thecal cells in ovaries from the control aged mice. The ovaries from açaí-treated aged mice showed this same trend with more robust expression. The ER stress-related protein PDIA4 was expressed in ovarian follicles from all three groups, but was localized to oocytes in the control aged mice only.

### 2.4. Alterations in Cellular Stress Pathways in the Oocyte

**Figure 5:**
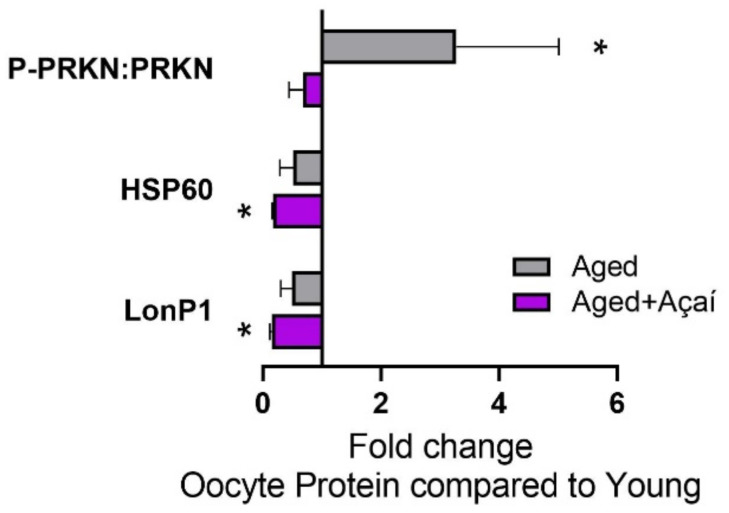
Effects of aging and açaí on oocyte signaling. Protein levels in the oocytes are presented as a fold change compared to young mice (n = 6 mice per group). The data were analyzed by the Kruskal–Wallis test with Dunn’s multiple comparisons when the overall effect *p* < 0.05. * *p* < 0.05 compared to young mice.

The activation of the mitophagy-initiating protein PRKN, as determined by the ratio of P-PRKN to total PRKN, was elevated threefold in control aged oocytes compared to young oocytes (*p* < 0.05), and this was prevented by açaí treatment (Figure 5).

The expression of the mitochondrial protein HSP60, which is important for the prevention of misfolded proteins, was lower in control aged oocytes compared to young oocytes, although not significantly (*p* = 0.30), and was lower in oocytes from açaí-treated aged mice (*p* < 0.05). The mitochondrial protease LonP1, which selectively degrades misfolded or oxidatively damaged proteins, showed the same pattern.

### 2.5. Molecular Effects of Aging and Açaí in the Uterus

**Figure 6:**
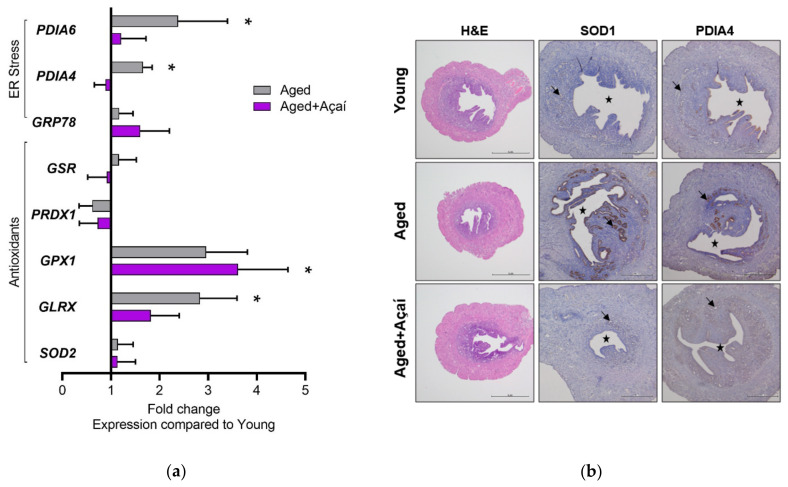
Uterine gene and protein expression. (**a**) Uterine tissue gene expression presented as a fold change compared to young mice (n = 10 per group). (**b**) Protein expression of SOD1 and PDIA4 in uterine tissue by immunohistochemistry. The black arrows point to glandular epithelium; the stars indicate the lumen surrounded by the luminal epithelium (n = 5 per group). Scale bars: 1 mm for the HE images and 500 μm for the IHC images. The gene expression data were analyzed by ordinary one-way ANOVA with Tukey’s multiple comparisons when the overall effect *p* < 0.05. * *p* < 0.05 compared to young mice.

The control aged mice showed increased uterine expression of the ER stress-related genes *PDIA4* and *PDIA6*, which was not observed following antioxidant supplementation (Figure 6a). Neither aging nor açaí affected antioxidant genes *GSR*, *PRDX1*, and *SOD2*, although *GPX1* and *GLRX* were significantly elevated in the açaí-treated and control aged mice, respectively (*p* < 0.05).

The levels of the antioxidant protein SOD1 and ER stress-response protein PDIA4 were higher in the glandular epithelium of control aged uteri compared to young uteri (Figure 6b). Açaí treatment prevented these aging-related changes.

## 3. Discussion

Diminished oocyte developmental competence associated with advanced maternal age is of primary concern for infertility medicine. Here, we report that açaí supplementation robustly improved oocyte quality to increase blastocyst development and implantation in an aged mouse model. Açaí exerted its effects by increasing the systemic antioxidant power and enacting molecular changes in the ovary, oocyte, and uterus. Namely, markers of ovarian ER stress and mitochondrial dysfunction that were elevated in the control aged mice were not detected in the açaí-treated aged mice.

Although the antioxidant treatment improved oocyte competence, it did not affect oocyte number. This is similar to what others have reported in animal models [29], and is what we expected based on our clinical observations [28]. Ovarian aging is associated with a loss of both the ovarian reserve and oocyte quality [2], and in this study we targeted the latter with a later-in-life antioxidant intervention.

Infertility patients of advanced maternal age often benefit from the use of donor oocytes [30]. Our data reflect this phenomenon, as we observed 87.1% implantation of embryos from young donors, but only 45.0% for embryos from aged donors, transferred to aged mice. Following açaí intervention, the donor embryos were of sufficient quality for 73.9% implantation in the control aged mice, nearing that of young donor embryos. These results reflect our clinical observations that regardless of maternal age, açaí supplementation prior to oocyte retrieval resulted in a high implantation rate following single euploid embryo transfer [28].

Protein and lipid biosynthesis, as well as the regulation of calcium homeostasis, take place in the ER, and are essential for the developing oocyte and early embryo. With aging in humans, oocytes have diminished ER function, which is associated with the accumulation of misfolded proteins and deficient calcium storage, which is detrimental to fertilization potential [31]. In both the whole ovary and the oocyte, we observed elevated levels of PDIA4, a member of the protein disulfide isomerase family that correlates with the intracellular ROS levels in the oocyte [14]. In addition, the gene expression of *GRP78*, an ER molecular chaperone that activates the UPR signaling cascade during ER stress, was higher in the control aged ovary than in the young or açaí-treated aged groups. These data indicate an activated ER stress response in the aged ovary that is not observed following açaí supplementation. Similar to our results, there are prior reports of ER stress modulated by antioxidants to improve oxidative stress-related pathologies, including PCOS and endometriosis [32,33], and to optimize oocyte in vitro maturation [34]. As ER stress and the accumulation of misfolded proteins can trigger apoptosis, these data are consistent with our preliminary report that açaí treatment in aged mice resulted in pro-survival and anti-apoptosis signaling compared to the control aged mice [28].

Our results revealed mitochondrial dysfunction in the aged ovary and oocyte. Mature oocytes have over 100,000 copies of mtDNA, with 2–10 copies per mitochondrion, representing the greatest number of mitochondria of any cell type [35]. Mitochondria are highly susceptible to persistent DNA damage due to their lack of nucleosomes and the proximity of ROS production to mtDNA, and mtDNA mutations, deletions, oxidative damage, and a reduced copy number are associated with aging [36,37]. We observed elevated oxidative DNA damage in the cytoplasm of oocytes from aged mice, indicating mtDNA damage, which was not present following açaí treatment. We suggest that base excision repair (BER), which is responsible for the removal of oxidized bases in mitochondria [38], was deficient in the ovaries and oocytes of aged mice, but was improved after antioxidant supplementation. This phenomenon can be explained by the ability of the redox system to directly modulate the activity of BER-associated enzymes [39,40,41].

There is evidence that defects in mitophagy contribute to mtDNA damage accumulation with aging [42]. However, we saw elevated DNA damage in the mitochondria of aged oocytes despite the increased activation of PRKN, a positive regulator of mitophagy. It is possible that mitophagy was not sufficient to outweigh the effects of oxidative damage; or, despite PRKN activation, mitophagic pathways were not adequately activated due to deficiencies in downstream factors such as autophagy-related (ATG) proteins [43]. The similar levels of PRKN activation in young and açaí-treated aged oocytes suggest that the mitochondrial quality was improved by açaí such that mitophagy was not triggered. The improved oocyte quality was likely not due to mitochondrial protein quality control, as the activation of the mitochondrial UPR—as determined by LonP1 and HSP60 protein levels—was blunted in aged oocytes, as previously observed [44], and this was not affected by antioxidants.

Mitochondrial dysfunction associated with aging results not only in further ROS production but also in less ATP production; indeed, aged oocytes have reduced ATP content, blunting their ability to develop properly [37,45,46]. As the mitochondria contained in oocytes represent those acquired by the embryo, poor mitochondrial quality contributes to reduced embryo development in aged mice. Conversely, improved mitochondrial quality following açaí treatment, i.e., reduced DNA damage and greater ATP production, likely represents a mechanism by which açaí improves embryo development and implantation potential.

NRF2 was slightly elevated in thecal and granulosa cells of ovarian follicles from aged mice, as is consistent with elevated oxidative stress [47], and was even higher in the açaí-treated aged mice. NRF2 is a “master regulator” of the antioxidant response, and it increases the transcription of many antioxidant enzymes [48]. Mitochondrial redox homeostasis and quality control, via mitophagy and mitochondrial biogenesis, are positively regulated by NRF2 as well [49]. Thus, high ovarian NRF2 levels following açaí treatment may be a mechanism for improved mitochondrial quality, and subsequently healthier ovarian follicles. Increased NRF2 in response to antioxidant supplementation has been reported in prior studies [50,51,52], and is likely a key mechanism by which ovarian function is improved by açaí treatment.

The investigation of inflammation-related signaling pathways in the ovary warrants future investigation, as the flavonoids in açaí have both anti-inflammatory and antioxidant properties [53]. Our preliminary transcriptome analysis of the ovary revealed the down-regulation of pro-inflammatory pathways following açaí supplementation in aged mice [28], but the role of these pathways in improved oocyte quality is yet undetermined.

Uterine signaling was disrupted in aged mice, but not in açaí-treated aged mice. The endometrial SOD1 expression was low in young mice, similar to prior observations in humans and animals [54,55]. Consistent with oxidative stress, SOD1 was highly expressed in aged mice; its expression was low in açaí-treated aged mice, similar to that in young mice. The same pattern was observed for *PDIA6* gene expression, as well as *PDIA4* gene and protein levels, indicating the activation of the ER stress response with aging. Aging-related aberrant endometrial levels of SOD1 and PDIA4 proteins occurred in the glandular epithelium, which secretes factors important for embryo implantation, stromal cell decidualization, and placentation [56]. We did not evaluate the effect of açaí treatment on the endometrium functionally; given its molecular effects, studying embryo transfer outcomes in açaí-treated aged recipients is warranted.

Based on the results of the current study and recent reports on antioxidant treatment improving the oxidative balance in the ovary [19,20,21], natural antioxidants are a promising strategy to combat reproductive aging [55]. Our data reflect improved oocyte competence via (1) reduced ROS through the direct scavenging activity of açaí, (2) the upregulation of NRF2, and (3) the prevention of cellular stress arising from oxidatively damaged biomolecule accumulation. Overall, açaí supplementation was observed to be a safe, effective strategy to improve ovarian function and subsequent oocyte quality.

## 4. Materials and Methods

### 4.1. Animals and Antioxidant Supplementation

Female outbred CF-1 mice (Charles River, Wilmington, MA, USA) were naturally aged in-house for 9 months prior to an 8-week dietary supplementation with 50 mg daily açaí administered in their drinking water. The açaí antioxidant supplement, sourced from a commercial manufacturer, contained only the naturally occurring açaí berry from the palm tree *Euterpe oleracea*. The açaí pulp underwent nonthermal dehydration and packaging into vegetable-based cellulose capsules (Ecofruits International Inc., South Jordan, UT, USA). The chemical analysis of the lot by the manufacturer reported a total polyphenol content of 6618 mg gallic acid equivalent (GAE)/100 g, an oxygen radical absorbance capacity (ORAC) of 208,628 μmol Trolox equivalent (TE)/100 g, and negligible microbial contamination. The aged control mice received the same standard chow diet, but without açaí supplementation. The young control mice, aged 8–12 weeks, also received the standard chow diet. The animal numbers differed for each experiment, as described below.

All of the mice were housed with a 12 h light/dark cycle and *ad libitum* access to a standard chow diet and water. The study was conducted according to the Guide for the Care and Use of Laboratory Animals (8th edition), and was approved by the Fertility Labs of Colorado Ethics in Research Committee.

### 4.2. Isolation of the Oocytes and Preimplantation Embryos

For the oocyte collection (n = 8 young, 14 aged, 12 aged + açaí), the mice were superovulated with 5 IU pregnant mare’s serum gonadotropin (PMSG; Sigma G-4877, St. Louis, MO, USA) followed 48 h later by 5 IU hCG (Sigma CG-5). The mice were euthanized by cervical dislocation 23 h after the hCG administration for oviduct dissection. The cumulus-oocyte complexes were isolated from the ampullae in G-MOPS+ (Vitrolife, Stockholm, Sweden) and were exposed to hyaluronidase (Sigma H-3757) to denude the cumulus cells. The oocytes were stored at −80 °C in PBS+PVA for later protein analysis.

The donor young, aged, and aged + açaí mice (n = 10, 20, and 12, respectively) underwent superovulation prior to mating with fertile CF-1 males. The female mice were superovulated with 5 IU PMSG, followed 48 h later by 5 IU hCG, then immediately bred overnight. A copulatory plug was taken as evidence of successful mating, and was considered D1 of pregnancy. On D4 of pregnancy, the mice were euthanized by cervical dislocation, and the uterine horns were dissected and flushed with G-MOPS+ (Vitrolife). The embryos were collected from the uterine flushings and allowed to equilibrate in G-2 Plus Media (Vitrolife) at 37 °C under 5% O_2_ and 7% CO_2_ prior to embryo transfer.

### 4.3. Embryo Transfer

The recipient aged mice (n = 19) were bred with vasectomized CF-1 males; a copulatory plug was taken as evidence of successful mating, and was considered D1 of pseudopregnancy. On D4 of pseudopregnancy, the recipient mice underwent embryo transfer under tribromoethanol anesthesia at 0.014 mL/g body weight. Through a dorsal incision, 3–6 blastocysts were transferred per uterine horn, proximal to the uterotubal junction. On D8, the recipient mice were euthanized by cervical dislocation, their uterine horns were excised, and the number of viable implantation sites was determined.

### 4.4. Serum DNA Damage and FRAP Assays

Whole blood was collected from non-pregnant female mice in proestrus, allowed to clot on ice for 15 min, and centrifuged at 16,000 rpm. The serum was stored at −80 °C.

For the determination of DNA damage, 8-hydroxy-2′-deoxyguanosine (8-OH-2′-dG) was measured in the serum, in duplicate, using a competitive enzyme-linked immunosorbent assay (ELISA) (Abcam #ab201734, Cambridge, UK) (n = 6 young, 8 aged, 8 aged + açaí). The serum was diluted 1:5 in Sample and Standard Diluent, and 50 μL diluted serum or standard was combined with 50 μL 8-OH-2′-dG HRP-conjugated monoclonal antibody. After 1 h at room temperature, the plate was rinsed with Wash Buffer four times. Then, 100 μL 3,3′,5,5′-tetramethylbenzidine (TMB) substrate was added, and the enzymatic color reaction developed in the dark at room temperature for 30 min. The reaction was stopped using 100 μL Stop Solution, and absorbance was read at 450 nm. Absorbance of the negative control well was subtracted from the test wells prior to the calculation of the average of the technical replicates, and the 8-OH-2′-dG concentrations were extrapolated from the standard curve.

The antioxidant capacity of the serum was determined in duplicate using the ferric reducing antioxidant power (FRAP) assay (Abcam #ab234626) (n = 6 young, 9 aged, 9 aged + açaí). The serum was diluted 1:2 in FRAP Assay Buffer, and a standard curve was prepared using a 2 mM Ferrous Standard and FRAP Assay Buffer. In total, 10 μL diluted serum or standards were combined with 152 μL FRAP Assay Buffer, 19 μL FRAP Probe, and 19 μL FeCl_3_ Solution, and then incubated at 37 °C for 60 min, then absorbance was read at 594 nm. The absorbance of the negative control well was subtracted from the test wells prior to the calculation of the average of the technical replicates, and FRAP values were extrapolated from the standard curve.

### 4.5. RNA Isolation and Quantitative Polymerase Chain Reaction

The ovaries and uterine tissue were harvested from non-pregnant female mice in proestrus (n = 10 per group) and sonicated in Norgen SKP Lysis Buffer, then the total RNA was isolated using the Norgen RNA/DNA/Protein Purification Plus Kit (Norgen #47700, Ontario, Canada) according to the manufacturer’s protocol. The RNA was quantified using NanoDrop 2000 (Thermo Fisher), then 2 μg of the total RNA was used for cDNA synthesis. The cDNA was synthesized using a High-Capacity cDNA Reverse Transcription Kit (Applied Biosystems #4368814, Foster City, CA, USA) according to the manufacturer’s protocol, then diluted 1:4 in Tris-EDTA buffer and stored at −20 °C. For the quantitative polymerase chain reaction (qPCR), 3 μL diluted cDNA, 0.3 μL 5 μM primer dilution, 4.2 μL nuclease-free reaction buffer, and 7.5 μL PowerSYBR Green PCR Master Mix (Applied Biosystems) were combined and subjected to amplification at 95 °C for 10 min, 40 cycles at 95 °C for 15 s, and 60 °C for 1 min, followed by a melt curve stage at 95 °C for 15 s, 60 °C for 1 min, and 95 °C for 15 s. The relative abundance to endogenous *18S* was utilized for the sample-to-sample comparisons, and the qPCR data were analyzed using the 2^−ΔΔCT^ method.

### 4.6. RNA In Situ Hybridization and Immunohistochemistry

The mice in proestrus were identified and euthanized by cervical dislocation for the collection of the ovaries and uterine horns (n = 10 per group). The tissues were incubated overnight in 4% paraformaldehyde, then paraffin-embedded and cut into 5-μm serial sections.

In order to localize the RNA expression of *GRP78* and *XBP1-S*, in situ hybridization was carried out according to the RNAscope Multiplex Fluorescent V2 Assay (Advanced Cell Diagnostics, Newark, CA, USA). The paraffin-embedded sections were de-paraffinized in xylene and 100% ethanol. RNAscope Hydrogen Peroxide Solution was applied at room temperature for 10 min, and then target retrieval was performed in RNAscope 1X Target Retrieval Reagent at 100 °C for 15 min. The proteins were denatured with RNAscope Protease Plus for 30 min at room temperature. Then, probes against *GRP78* (channel 3; #438831-C3) and *XBP1-S* (channel 1; #413541) were hybridized to the sections for 2 h at 40 °C. The amplification molecules were each hybridized for 30 min at 40 °C. The horseradish peroxidase (HRP) signals for channels 1 and 3 were developed for 15 min at 40 °C, followed by the corresponding Opal fluorophores (Akoya Biosciences, Marlborough, MA, USA) for 15 min at 40 °C: Opal 570 Reagent for channel 1 and Opal 690 Reagent for channel 3. ProLong Gold Antifade Mountant with DAPI (Thermo Fisher) and coverslips were applied, and the slides were stored at 4 °C in the dark.

For the immunohistochemistry, the paraffin-embedded sections were de-paraffinized in xylene and rehydrated in decreasing concentrations of ethanol (100–50%) and water. The antigen retrieval was carried out in 10 mM, pH 6.0 sodium citrate buffer at 100 °C for 15 min. The endogenous enzymes were quenched with 0.3% hydrogen peroxide in TBS for 20 min. The sections were blocked in 1% bovine serum albumin (BSA) and 10% normal goat serum in TBS for 45 min. The primary antibodies were diluted in 1% BSA in TBS and incubated at 4 °C overnight (8-OH-2′-dG, 1:200, Bioss BS-1278R, Woburn, MA, USA; NRF2, 1:500, Novus NBP1-32822, Centennial, CO, USA; PDIA4, 1:500, Abcam ab155800; SOD1, 1:500, Abcam ab51254). The sections were incubated with secondary anti-HRP antibody (1:10,000) in 1% BSA in TBS for 1 h. DAB substrate (Dako, Santa Clara, CA, USA) was added and developed for 10 min, or until a brown color was visible. The sections were counterstained with hematoxylin for 10 sec prior to dehydration with increasing concentrations of ethanol (70–100%) and xylene. The staining was preserved with Limonene mounting media (Abcam) and cover slips.

### 4.7. Jess Simple Western Blotting

Simple Western 12–230 kDa size assays were carried out using a Jess analyzer (Protein Simple, San Jose, CA, USA). This automated Western blotting approach uses capillary electrophoresis to detect the protein of interest. Three MII oocytes per mouse were used to detect HSP60 (R&D Systems AF1800-SP, Minneapolis, MN, USA) and LONP1 (Novus Biologicals NBP1-81734), and 10 oocytes per mouse were used to detect the phosphorylated (ser 65) PRKN (Biorbyt orb312554, St. Louis, MO, USA) and total PRKN (Thermo Fisher 21H24L9). Six mice per group were used for the analysis. Post thawing, the oocytes were lysed in RIPA buffer (Sigma-Aldrich) supplemented with protease and phosphatase inhibitors (Sigma-Aldrich). A Simple Western Jess immunoassay was then performed as previously described [56]. After the assay’s completion, the results were generated as electropherograms by Compass for Simple Western software. A clear peak at the associated molecular weight indicated the presence of the target protein, and the area under the peak was used for the protein quantification. The target protein expression was normalized to the total protein signal from the same capillary. Bar graphs were then generated using the fold changes relative to the controls.

### 4.8. Statistical Analysis

The continuous data were compared by ordinary one-way analysis of variance (ANOVA) with Tukey’s multiple comparisons, or the Kruskal–Wallis test with Dunn’s multiple comparisons, where appropriate. For the implantation rate comparisons, the data were analyzed by Fisher’s exact test with the Koopman asymptotic score to determine the relative rates.

## References

[1] Keefe D., Kumar M., Kalmbach K. (2015). Oocyte competency is the key to embryo potential. Fertil. Steril..

[2] Llarena N., Hine C. (2020). Reproductive Longevity and Aging: Geroscience Approaches to Maintain Long-Term Ovarian Fitness. J. Gerontol. Ser. A Boil. Sci. Med. Sci..

[3] Martin J.A., E Hamilton B., Osterman M.J.K., Driscoll A.K. (2019). Births: Final Data for 2018. Natl. Vital Stat. Rep..

[4] La Marca A., Capuzzo M., Imbrogno M.G., Donno V., Spedicato G.A., Sacchi S., Minasi M.G., Spinella F., Greco P., Fiorentino F. (2021). The complex relationship between female age and embryo euploidy. Minerva Obstet. Gynecol..

[5] Greaney J., Wei Z., Homer H. (2017). Regulation of chromosome segregation in oocytes and the cellular basis for female meiotic errors. Hum. Reprod. Updat..

[6] Van Blerkom J. (2011). Mitochondrial function in the human oocyte and embryo and their role in developmental competence. Mitochondrion.

[7] Lord T., Aitken R.J. (2013). Oxidative stress and ageing of the post-ovulatory oocyte. Reproduction.

[8] Lim J., Luderer U. (2011). Oxidative Damage Increases and Antioxidant Gene Expression Decreases with Aging in the Mouse Ovary. Biol. Reprod..

[9] Ruder E.H., Hartman T.J., Blumberg J., Goldman M.B. (2008). Oxidative stress and antioxidants: Exposure and impact on female fertility. Hum. Reprod. Updat..

[10] Wang S., Zheng Y., Li J., Yu Y., Zhang W., Song M., Liu Z., Min Z., Hu H., Jing Y. (2020). Single-Cell Transcriptomic Atlas of Primate Ovarian Aging. Cell.

[11] Fujii J., Iuchi Y., Okada F. (2005). Fundamental roles of reactive oxygen species and protective mechanisms in the female reproductive system. Reprod. Biol. Endocrinol..

[12] Freitas C., Neto A.C., Matos L., Silva E., Ribeiro Â., Silva-Carvalho J.L., Almeida H. (2017). Follicular Fluid redox involvement for ovarian follicle growth. J. Ovarian Res..

[13] Bianchi S., Macchiarelli G., Micara G., Linari A., Boninsegna C., Aragona C., Rossi G., Cecconi S., Nottola S.A. (2015). Ultrastruc tural markers of quality are impaired in human metaphase II aged oocytes: A comparison between reproductive and in vitro aging. J. Assist. Reprod. Genet..

[14] Yuan Y., Wheeler M., Krisher R.L. (2012). Disrupted Redox Homeostasis and Aberrant Redox Gene Expression in Porcine Oocytes Contribute to Decreased Developmental Competence1. Biol. Reprod..

[15] Zorov D.B., Juhaszova M., Sollott S.J. (2014). Mitochondrial Reactive Oxygen Species (ROS) and ROS-Induced ROS Release. Physiol. Rev..

[16] Ray P.D., Huang B.-W., Tsuji Y. (2012). Reactive oxygen species (ROS) homeostasis and redox regulation in cellular signaling. Cell. Signal..

[17] Poprac P., Jomova K., Simunkova M., Kollar V., Rhodes C.J., Valko M. (2017). Targeting Free Radicals in Oxidative Stress-Related Human Diseases. Trends Pharmacol. Sci..

[18] García-Sánchez A., Miranda-Díaz A.G., Cardona-Muñoz E.G. (2020). The Role of Oxidative Stress in Physiopathology and Pharmacological Treatment with Pro- and Antioxidant Properties in Chronic Diseases. Oxidative Med. Cell. Longev..

[19] Espino J., Macedo M., Lozano G., Ortiz Á., Rodríguez C., Rodríguez A.B., Bejarano I. (2019). Impact of Melatonin Supplementation in Women with Unexplained Infertility Undergoing Fertility Treatment. Antioxidants.

[20] Giannubilo S.R., Orlando P., Silvestri S., Cirilli I., Marcheggiani F., Ciavattini A., Tiano L. (2018). CoQ10 Supplementation in Patients Undergoing IVF-ET: The Relationship with Follicular Fluid Content and Oocyte Maturity. Antioxidants.

[21] Rodríguez-Varela C., Labarta E. (2021). Does Coenzyme Q10 Supplementation Improve Human Oocyte Quality?. Int. J. Mol. Sci..

[22] Xu Y., Nisenblat V., Lu C., Li R., Qiao J., Zhen X., Wang S. (2018). Pretreatment with coenzyme Q10 improves ovarian response and embryo quality in low-prognosis young women with decreased ovarian reserve: A randomized controlled trial. Reprod. Biol. Endocrinol..

[23] Earling M., Beadle T., Niemeyer E.D. (2019). Açai Berry (*Euterpe oleracea*) Dietary Supplements: Variations in Anthocyanin and Flavonoid Concentrations, Phenolic Contents, and Antioxidant Properties. Plant Foods Hum. Nutr..

[24] Dattilo M., D’Amato G., Caroppo E., Ménézo Y. (2016). Erratum to: Improvement of gamete quality by stimulating and feeding the endogenous antioxidant system: Mechanisms, clinical results, insights on gene-environment interactions and the role of diet. J. Assist. Reprod. Genet..

[25] Barbosa P.O., Souza M.O., Silva M.P., Santos G.T., Silva M.E., Bermano G., Freitas R.N. (2021). Açaí (*Euterpe oleracea* Martius) supplementation improves oxidative stress biomarkers in liver tissue of dams fed a high-fat diet and increases antioxidant enzymes’ gene expression in offspring. Biomed. Pharmacother..

[26] Vilhena J.C., Lopes de Melo Cunha L., Jorge T.M., de Lucena Machado M., de Andrade Soares R., Santos I.B., Freitas de Bem G., Fernandes-Santos C., Ognibene D.T., Soares de Moura R. (2021). Açaí Reverses Adverse Cardiovascular Remodeling in Renovascular Hypertension: A Comparative Effect with Enalapril. J. Cardiovasc. Pharmacol..

[27] Monteiro C., Filho H., Silva F.G.O., de Souza M.F.F., Sousa J.A.O., Franco Á X., Resende Â C., de Moura R.S., de Souza M.H.L., Soares P.M.G. (2021). *Euterpe oleracea* Mart. (Açaí) attenuates experimental colitis in rats: Involvement of TLR4/COX-2/NF-ĸB. Inflammopharmacology.

[28] Katz-Jaffe M.G., Lane S.L., Parks J.C., McCallie B.R., Makloski R., Schoolcraft W.B. (2020). Antioxidant Intervention Attenuates Aging-Related Changes in the Murine Ovary and Oocyte. Life.

[29] Budani M.C., Tiboni G.M. (2020). Effects of Supplementation with Natural Antioxidants on Oocytes and Preimplantation Embryos. Antioxidants.

[30] Kawwass J.F., Monsour M., Crawford S., Kissin D.M., Session D.R., Kulkarni A.D., Jamieson D., National ART Surveillance System (NASS) Group (2013). Trends and outcomes for donor oocyte cycles in the United States, 2000–2010. JAMA.

[31] Igarashi H., Takahashi E., Hiroi M., Doi K. (1997). Aging-related changes in calcium oscillations in fertilized mouse oocytes. Mol. Reprod. Dev..

[32] Brenjian S., Moini A., Yamini N., Kashani L., Faridmojtahedi M., Bahramrezaie M., Khodarahmian M., Amidi F. (2019). Resveratrol treatment in patients with polycystic ovary syndrome decreased pro-inflammatory and endoplasmic reticulum stress markers. Am. J. Reprod. Immunol..

[33] Lin X., Dai Y., Tong X., Xu W., Huang Q., Jin X., Li C., Zhou F., Zhou H., Lin X. (2020). Excessive oxidative stress in cumulus granulosa cells induced cell senescence contributes to endometriosis-associated infertility. Redox Biol..

[34] Lin T., Lee J.E., Kang J.W., Shin H.Y., Bin Lee J., Jin D.I. (2019). Endoplasmic Reticulum (ER) Stress and Unfolded Protein Response (UPR) in Mammalian Oocyte Maturation and Preimplantation Embryo Development. Int. J. Mol. Sci..

[35] May-Panloup P., Boucret L., De La Barca J.-M.C., Desquiret-Dumas V., Ferré-L’Hotellier V., Morinière C., Descamps P., Procaccio V., Reynier P. (2016). Ovarian ageing: The role of mitochondria in oocytes and follicles. Hum. Reprod. Updat..

[36] Chocron E.S., Munkácsy E., Pickering A.M. (2018). Cause or casualty: The role of mitochondrial DNA in aging and age-associated disease. Biochim. Biophys. Acta Mol. Basis Dis..

[37] Simsek-Duran F., Li F., Ford W., Swanson R.J., Jones H.W., Castora F.J. (2013). Age-Associated Metabolic and Morphologic Changes in Mitochondria of Individual Mouse and Hamster Oocytes. PLoS ONE.

[38] Sykora P., Kanno S., Akbari M., Kulikowicz T., Baptiste B.A., Leandro G.S., Lu H., Tian J., May A., Becker K.A. (2017). DNA Polymerase Beta Participates in Mitochondrial DNA Repair. Mol. Cell. Biol..

[39] Kelley M.R., Parsons S.H. (2001). Redox regulation of the DNA repair function of the human AP endonuclease Ape1/ref-1. Antioxid. Redox Signal.

[40] Bravard A., Vacher M., Moritz E., Vaslin L., Hall J., Epe B., Radicella J.P. (2009). Oxidation Status of Human OGG1-S326C Polymorphic Variant Determines Cellular DNA Repair Capacity. Cancer Res..

[41] Cuneo M.J., London R.E. (2010). Oxidation state of the XRCC1 N-terminal domain regulates DNA polymerase beta binding affinity. Proc. Natl. Acad. Sci. USA.

[42] Gaziev A.I., Abdullaev S., Podlutsky A. (2014). Mitochondrial function and mitochondrial DNA maintenance with advancing age. Biogerontology.

[43] Ding W.X., Yin X.M. (2012). Mitophagy: Mechanisms, pathophysiological roles, and analysis. Biol. Chem..

[44] Pollecker K., Sylvester M., Voos W. (2021). Proteomic analysis demonstrates the role of the quality control protease LONP1 in mitochondrial protein aggregation. J. Biol. Chem..

[45] Van Blerkom J., Davis P.W., Lee J. (1995). Fertilization and early embryolgoy: ATP content of human oocytes and developmental potential and outcome after in-vitro fertilization and embryo transfer. Hum. Reprod..

[46] Igarashi H., Takahashi T., Takahashi E., Tezuka N., Nakahara K., Takahashi K., Kurachi H. (2005). Aged Mouse Oocytes Fail to Readjust Intracellular Adenosine Triphosphates at Fertilization1. Biol. Reprod..

[47] Wang L., Tang J., Wang L., Tan F., Song H., Zhou J., Li F. (2021). Oxidative stress in oocyte aging and female reproduction. J. Cell. Physiol..

[48] Shaw P., Chattopadhyay A. (2019). Nrf2–ARE signaling in cellular protection: Mechanism of action and the regulatory mechanisms. J. Cell. Physiol..

[49] Ryoo I.G., Kwak M.K. (2018). Regulatory crosstalk between the oxidative stress-related transcription factor Nfe2l2/Nrf2 and mitochondria. Toxicol. Appl. Pharmacol..

[50] Chen S., Lu Y., Chen Y., Xu J., Chen L., Zhao W., Wang T., Wang H., Wang P. (2021). The effect of Bu Shen Huo Xue Tang on autoimmune premature ovarian insufficiency via Modulation of the Nrf2/Keap1 signaling pathway in mice. J. Ethnopharmacol..

[51] Liu X., Lin X., Zhang S., Guo C., Li J., Mi Y., Zhang C. (2018). Lycopene ameliorates oxidative stress in the aging chicken ovary via activation of Nrf2/HO-1 pathway. Aging.

[52] Wang Y., Li N., Zeng Z., Tang L., Zhao S., Zhou F., Zhou L., Xia W., Zhu C., Rao M. (2021). Humanin regulates oxidative stress in the ovaries of polycystic ovary syndrome patients via the Keap1/Nrf2 pathway. Mol. Hum. Reprod..

[53] Odendaal A.Y., Schauss A.G., Watson R.R., Preedy V.R., Zibadi S. (2014). Chapter 18—Potent Antioxidant and Anti-Inflammatory Flavonoids in the Nutrient-Rich Amazonian Palm Fruit, Açaí (*Euterpe* spp.). Polyphenols in Human Health and Disease.

[54] Sugino N., Shimamura K., Takiguchi S., Tamura H., Ono M., Nakata M., Nakamura Y., Ogino K., Uda T., Kato H. (1996). Changes in activity of superoxide dismutase in the human endometrium throughout the menstrual cycle and in early pregnancy. Hum. Reprod..

[55] Santos C., Pires M.D.A., Santos D., Carreira R.P. (2016). Distribution of superoxide dismutase 1 and glutathione peroxidase 1 in the cyclic canine endometrium. Theriogenology.

[56] Kelleher A., DeMayo F.J., E Spencer T. (2019). Uterine Glands: Developmental Biology and Functional Roles in Pregnancy. Endocr. Rev..

